# Reject rate analysis in digital radiography: an Australian emergency imaging department case study

**DOI:** 10.1002/jmrs.343

**Published:** 2019-07-18

**Authors:** Samantha Atkinson, Michael Neep, Deborah Starkey

**Affiliations:** ^1^ South Coast Radiology Pindara Private Hospital Benowa Queensland Australia; ^2^ Department of Medical Imaging Logan Hospital Meadowbrook Queensland Australia; ^3^ School of Public Health and Social Work Queensland University of Technology Brisbane Australia; ^4^ Institute of Health and Biomedical Innovation Queensland University of Technology Brisbane Australia; ^5^ School of Clinical Sciences Queensland University of Technology Brisbane Australia

**Keywords:** Computed radiography, digital radiography, reject analysis, reject rate

## Abstract

**Introduction:**

Reject analysis in digital radiography (DR) helps guide the education and training of staff, influences department workflow, reduces patient dose and improves department efficiency. The purpose of this study was to investigate rejected radiographs at a major metropolitan emergency imaging department to help form a benchmark of reject rates for DR and to assess what radiographs are being rejected and why.

**Methods:**

A retrospective longitudinal study was undertaken as an in‐depth clinical audit. The data were collected using automated reject analysis software from two digital x‐ray systems from June 2015 to April 2017. The overall reject rate, reasons for rejection as well as the reject rates for individual radiographers, examination types and projections were analysed.

**Results:**

A total of 90,298 radiographic images were acquired and included in the analysis. The average reject rate was 9%, and the most frequent reasons for image rejection were positioning error (49%) and anatomy cut‐off (21%). The reject rate varied between radiographers as well as for individual examination types and projections.

**Conclusions:**

The variation in radiographer reject rates and the high reject rate for some projections indicate that reject analysis is still necessary as a quality assurance tool for DR. A feedback system between radiologists and radiographers may reduce the high percentage of positioning errors by standardising the technical factors used to assess image quality. Future reject analysis should be conducted regularly incorporating an exposure indicator analysis as well as retrospective assessment of individual rejected images.

## Graphical abstract

Reject analysis in radiography is a valuable quality assurance tool that was used frequently in plain film and computed radiography to monitor patient dose, image quality and department efficiency. This in‐depth clinical audit assessed 90,298 images acquired in a digital emergency imaging department from June 2015 to April 2017 with the aim of identifying a benchmark reject rate for digital radiography and to investigate what images were being rejected more frequently and why. The average reject rate was 9%; however, this varied significantly between individual projections as well as individual radiographers, highlighting the need to continue to incorporate reject analysis into a standard quality assurance program for digital radiography.

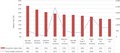

## Introduction

Reject analysis is an important component of quality assurance programs for medical imaging departments. It forms a basis for determining the causes of rejected images and helps guide radiographer training, department workflow and ultimately reduces patient dose.^1^, ^2^, ^3^, ^4^, ^5^ The objective of a radiographic examination was to acquire diagnostic images in at least two planes to help diagnose conditions or injuries while minimising the exposure to ionising radiation that the patient receives. A rejected image is defined as a radiograph that is deemed unacceptable in terms of image quality, by the radiographer at the time of acquisition.^3^, ^6^ The radiographer makes the judgement that an image does not satisfy stringent technical qualities to contribute to the definitive diagnosis and subsequently rejects the image and is therefore required to take another. This repeat imaging increases the patient's radiation dose and detracts from the principle of keeping the patient's exposure to ionising radiation to ‘As Low As Reasonably Achievable’ (ALARA).^3^, ^7^ In addition, rejected images decrease department efficiency and patient satisfaction, consequently increasing departmental expenses.^7^

Without reject analysis, it is difficult to keep track of the number of rejected and repeated images and in some instances, there may be no evidence of a rejected image. Consequently, it is difficult to control and reduce the reject rate. This detracts from a medical imaging department's commitment to radiation safety. The Australian Code of Conduct for medical radiation practitioners states that it is the responsibility of the medical radiation practitioner (the radiographer) to promote the safe use of radiation.^8^ This includes justifying, limiting and optimising each exposure, while still acquiring quality diagnostic images in accordance with the ALARA principle.^8^ Reject analysis helps to promote the safe use of radiation by monitoring the ionising radiation delivered to patients and the quality of the images produced and should therefore be a standard component of quality assurance programs.

It was expected that reject rates would decrease with the introduction of digital radiography (DR).^1^ This was because of the improved image quality available with DR as a result of the increased exposure latitude and post‐processing capabilities.^1^, ^4^ International studies, however, have reported DR reject rates that are higher than those reported for computed radiography (CR).^1^, ^2^, ^4^ The reject rates reported for CR were around 5%.^2^, ^3^ Departments with a combination of CR and DR are now reporting reject rates ranging from 4% to 11%.^1^, ^5^

Most Australian medical imaging departments are now solely using DR equipment, due to incentives offered by the Australian government through the Medicare Benefits Schedule (capital sensitivity and financial rebates).^9^ However, there is a paucity of published articles that are specific to DR reject rates that have utilised a large sample size and there have been no such studies conducted in Australia. Subsequently, Australian medical imaging departments are unable to compare their DR reject rates to an equivalent Australian evidence‐base to determine whether their reject rates are appropriate. This has implications for monitoring the effectiveness, safety and diagnostic quality of contemporary radiographic practice.^9^

Reject analysis with most DR and CR systems is now a simple, efficient process that utilises automated reject data collection and analysis software. Previously with conventional film‐screen radiography, reject analysis was undertaken manually by collecting, sorting and then analysing the physical films.^10^, ^11^, ^12^ The need to develop automated reject analysis software was identified soon after CR implementation and two studies piloted such software.^4^, ^13^ Later studies tested and utilised automatic reject analysis software, which is now widely available on most CR and DR systems.^2^, ^3^, ^14^

The reasons for image rejection have changed in accordance with technological advancements. The most frequent reason for image rejection with conventional film‐screen radiography was exposure error (i.e. over exposure or under exposure). This is now reported as positioning errors with CR and DR.^3^, ^4^, ^5^, ^10^, ^15^ The accuracy of the results from a reject analysis study rely on the compliance of radiographers to categorise their rejected images correctly.^10^ Furthermore, the ability of an imaging department to reduce their reject rate relies on the application of reject analysis findings through the development of a regular feedback and education system.

The aim of this study was to report the reject rate for DR at a major Australian metropolitan emergency imaging department (44,679 x‐rays per annum in 2016). It investigated why images were being rejected, what type of examinations and projections were rejected more frequently, and how the reject rate varied between different radiographers.

## Method

### Ethics

Exemption from a full ethical review was approved from the Human Research Ethics Committees from the Metro South Health Human Research Ethics Committee (HREC), and the Queensland University of Technology (QUT) Office of Research Ethics and Integrity (OREI). Informed Consent was not required as all data were accumulated retrospectively and de‐identified.

### Design

A retrospective longitudinal study of data collected from June 2015 to April 2017 was undertaken as part of an in‐depth clinical audit.

### Setting

The data were collected from a major metropolitan emergency medical imaging department in Meadowbrook, Queensland, Australia (*n* = 44,679 x‐rays per annum in 2016). The emergency medical imaging department consists of three fixed DR rooms (two Agfa and one Philips), an orthopantomogram (OPG) machine, and a DR mobile machine. All images acquired in either of the two DR Agfa rooms were included, while images acquired in the third x‐ray room, as well as mobile and OPG examinations were excluded. The data gathered in the third x‐ray room were excluded from the study as it was not a main x‐ray room and the data collection software was unique to the Philips manufacturer and therefore used disparate terminology to categorise rejected images. The two included DR workstations were both ceiling mounted DR systems (Agfa DXD 600) that consisted of one large (35 × 43 cm) and one small (30 × 40 cm) wireless DR detector, and a fixed vertical detector (43 × 43 cm). One room had a fixed table detector (43 × 43 cm) whereas the other had a Bucky tray.

### Procedure

The data were collected using automated reject data collection and analysis software, which came as a standard component of the DR system. This software recorded all images that were accepted and rejected on the workstation. When an image was rejected, before another image could be taken, the radiographer was prompted to select a reason for rejection from a drop‐down list (Table 1).

**Table 1:** The reasons for image rejection available for radiographers to choose from

Positioning	Test
Anatomy cut‐off	Inappropriate image processing
Artefact	Software failure
No image	Mechanical failure
Patient movement	Other failure
Under exposed	Over exposed
Poor inspiration	Electrical failure

For each image acquired in the two x‐ray rooms, the automatic reject data collection software recorded several criteria (Table 2). An authorised user manually exported these data into spreadsheets on an encrypted hard‐drive. Once exported, the data from each workstation were combined to create one data set and some filtering was undertaken to simplify the data (e.g. the radiographers were anonymised, the date and time stamps were reformatted, and the terminology was assessed to ensure uniformity). The original data were then automatically deleted from the workstation to allow for more storage space.

**Table 2:** Criteria recorded for each image acquired on each workstation

Criteria	Definition
Hospital	The site where the workstation is located.
Department	The specific department where the workstation is located, such as emergency room one or emergency room two.
Exam group	The exam group as specified on the workstation.
Exposure type	The specific projection as specified on the workstation, such as oblique or lateral.
Body part	The general body part as specified by the Radiology Information System (RIS) system (less specific than the exam group).
Acquisition date and time	The date and time of image acquisition.
Reject reason	The reason for image rejection as selected by the radiographer. If the image was accepted, this section remains blank.
Instance identifier number	An identifier number specific to a single image.
Session identifier number	An identifier number specific to a single patient examination, which may consist of multiple instance identifier numbers.
Reject status	Each image is categorised as one of: rejected, accepted or unrejected.
Operator	The radiographer's initials entered at the time of image acquisition. It is possible to enter multiple initials.

### Data analysis

Secondary data analysis of the raw data was completed using standard descriptive statistics on Microsoft Excel and IBM SPSS Statistics 23. The average reject rate was calculated by dividing the total number of rejected images by the total number of images acquired and expressed as a percentage, along with the standard deviation. The data were filtered, the mean and standard deviation were calculated and the results were expressed as percentages in order to assess the reasons for image rejection, the reject rate for each examination type and projection, as well as individual radiographer reject rates.

## Results

### Sample size and exclusions

An overall sample size of 90,298 images, collected from 32,491 examinations over 15 months from June 2015 to April 2017 was identified as reliable data that met the inclusion criteria for analysis. The automated data collection software records all of the images acquired on the workstation and is therefore a robust method of data collection.

### Reject rate

**Figure 1:**
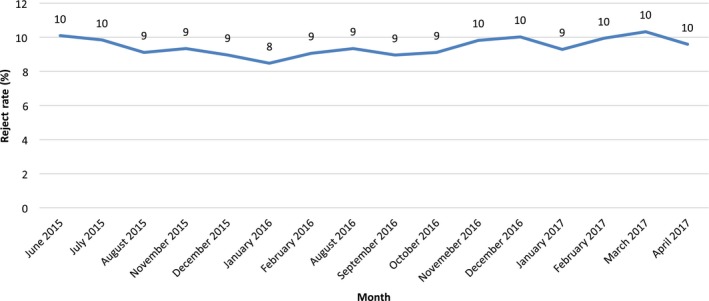
The average monthly reject rate for the emergency imaging department as recorded from June 2015 to April 2017. Note. Data for some months is not available due to a corrupt archive and subsequent missing data.

Of the 90,298 images acquired, 8578 were rejected, resulting in an average reject rate of 9% (SD = 1). Figure 1 demonstrates that the monthly reject rate was relatively constant throughout the study period, ranging from 8% to 10%.

### Reasons for image rejection

The identified reasons for rejection (*n*, reject rate) are displayed in Table 3. The most frequently reported reasons for image rejection were ‘positioning’ error (4207 images, 49%) and ‘anatomy cut‐off’ (1829 images, 21%). The number of images rejected for ‘under exposure’ represented 5% (412 images) of all rejected images, whereas images rejected for ‘over exposure’ represented 0.2% (17 images) of all rejected images.

**Table 3:** The identified reasons for image rejection

Reject reason	*n* (%)
Positioning	4207 (49%)
Anatomy cut‐off	1829 (21%)
Artefact	682 (8%)
No image	488 (6%)
Patient movement	416 (5%)
Under exposed	412 (5%)
Poor Inspiration	185 (2%)
Test	153 (2%)
Inappropriate image processing	70 (1%)
Software failure	56 (1%)
Mechanical failure	45 (1%)
Other failure	17 (0%)
Over exposed	17 (0%)
Electrical failure	5 (0%)
Total	8582

### Radiographer reject rates

Individual radiographers were anonymised and the top 20 radiographers who took the majority of images over the data collection period were analysed (Table 4). These data indicate that there are considerable variation in the reject rates between individual radiographers (Mean = 9%, SD = 3). The radiographers included in the study represented various levels of experience and utilised their own personal assessment of image quality based on both technical and diagnostic qualities.

**Table 4:** The reject rates of the individual radiographers (anonymised) that acquired the highest number of radiographic images over the data collection period

Radiographer	Rejected	Total images acquired	Reject rate (%)
A	363	4233	9
B	509	3036	17
C	258	2661	10
D	251	2637	10
E	241	2609	9
F	160	2497	6
G	255	2331	11
H	314	2206	14
I	146	2042	7
J	210	2023	10
K	227	2006	11
L	211	1887	11
M	142	1763	8
N	165	1734	10
O	175	1667	10
P	125	1665	8
Q	230	1535	15
R	74	1482	5
S	146	1440	10
T	136	1379	10

### Reject rates per examination type

There were 21 different types of examinations and the reject rate for each are displayed in Table 5. The frequency of rejected images for each examination type indicate that a number of examination types have reject rates considerably higher than the average reject rate of 9% (*n* = 13, Mean = 13%, SD = 6).

**Table 5:** The reject rates for each examination type

Examination type	Rejected	Total images acquired	Reject rate (%)
Abdomen	290	2490	12
Chest	2852	39,185	7
Cervical spine	369	2018	18
Thoracic spine	110	764	14
Lumbosacral spine	314	1722	18
Pelvis	496	2132	23
Hip	392	1671	23
Femur	91	609	15
Knee	948	4965	19
Tibia	110	1775	6
Ankle/calcaneus	564	6525	9
Foot/toes	209	7080	3
Lower limb	19	174	11
Shoulder/clavicle	621	4198	15
Humerus	61	451	14
Elbow	418	2958	14
Forearm	260	3259	8
Wrist	237	3008	8
Hand/fingers	182	4991	4
Upper limb	15	180	8
Skull/facial bones	23	160	14

### Reject rate per projection

**Figure 2:**
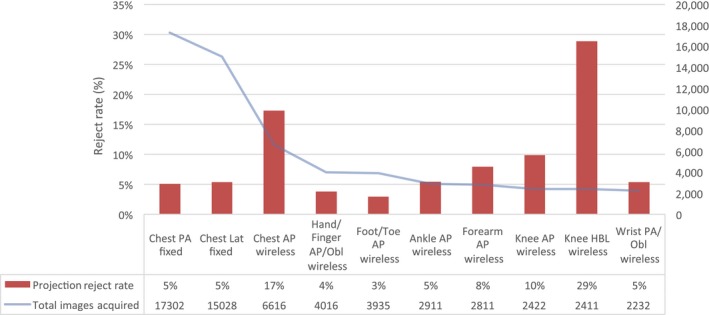
The reject rates for the 10 most frequently acquired projections with reference to the total images acquired for each projection. Note. PA, posterior anterior; LA, lateral; AP, anterior posterior; Obl, oblique; HBL, horizontal beam lateral.

The reject rate for the 10 most frequently acquired projections are presented in Figure 2. The posteroanterior (PA) and lateral chest projections acquired using the fixed vertical detector make up 19% and 17% of the total images acquired in the emergency imaging department respectively and have reject rates of 5% (PA Chest: 873 rejected/17302 acquired, lateral Chest: 803 rejected/15028 acquired). This is 4% below the average reject rate for the department. The anteroposterior (AP) chest projection acquired using the wireless detector is the third most frequently acquired projection, representing 7% of all images acquired in the emergency imaging department, and had a high reject rate of 17% (1142 rejected/6616 acquired).

**Figure 3:**
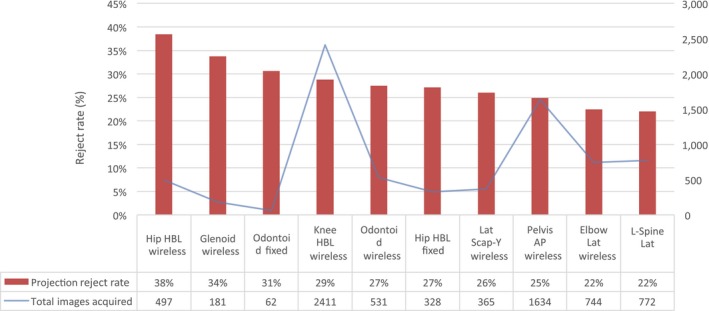
The reject rates of the 10 projections that represent the highest reject rates in this study with reference to the total images acquired for each projection. Note. HBL, horizontal beam lateral; LA, lateral; AP, anterior posterior.

Figure 3 demonstrates the projections that have the highest reject rates and identifies some trends. The horizontal beam lateral (HBL) hip projection acquired using a wireless detector is the highest rejected projection with a reject rate of 38%; however, it represents only 0.6% (497 images) of the total images acquired in the emergency imaging department. HBL knee projections using a wireless detector and HBL hip projections using the fixed vertical detector are also within the 10 highest rejected projections.

## Discussion

This was the first Australian‐based study to investigate DR reject rates, to the full exclusion of CR. This was a robust reject analysis with a large data set of 90, 298 images, which resulted in an average reject rate of 9%. Interestingly, when rejected images of individual projections were analysed, some anatomical regions yielded reject rates as high as 17%. This highlights a potential area of concern. Additional training that focuses on these specific projections may prove beneficial to reducing departmental reject rates and decreasing patient dose. It was also found that the image quality standards between radiographers may be inconsistent and could be improved with regular feedback so that the image quality and technical factors perceived as diagnostic by one radiographer are the same as another radiographer and radiologist. The purpose of a reject analysis is to identify areas within the department that require optimisation. This study therefore identified limitations in both department performance as well as the current method of reject analysis allowing recommendations to be made to help improve performance and to strengthen future reject analysis.

Rejected images are not sent to the radiologist for diagnosis; subsequently, a high reject rate has implications for patient dose and department performance.^4^ The average reject rate for DR reported by two international studies was 4% and 11%.^1^, ^5^ Both of these studies analysed reject rates only in general radiography and although the first study had a large sample size of 98,503 images, they were both conducted over less than a 6‐month period. The reject rate found in this study was 9% and is towards the higher end of reject rates reported for DR. This reject rate is also higher than the average of 5% reported for CR^2^, ^3^, ^4^, ^10^, ^11^ and is comparable to film‐screen studies, which ranged from 8% to 16%.^6^, ^10^, ^11^ The results from this study however are a more accurate reflection of reject rates for emergency radiography to the full exclusion of general radiography (radiography performed outside of the emergency department).

A number of examination types had reject rates considerably higher than the overall average. These results support the findings of Dunn and Rogers,^6^ who found that reject rates are sensitive to examination type and that using a single average reject rate as an indicator of quality has the potential to be misrepresentative of actual performance. For example, this study found that the projection with the highest reject rate was the horizontal beam lateral (HBL) hip at 38%, and the HBL knee projection represented the fourth most frequently rejected projection with a reject rate of 29%. Both of these projections require more challenging radiographic techniques. Likewise, the AP chest and AP pelvis projections acquired using a wireless detector had reject rates higher than the average and higher than the comparable projections acquired using a fixed detector. Again, these modified projections are more challenging in terms of patient position, detector and tube alignment as well as exposure adjustments. Furthermore, the average reject rate for the entire department is skewed by projections with significantly larger sample sizes and lower reject rates such as the PA and lateral chest, and basic extremity projections. Analysis of the reject rates for individual examination types and projections allows in‐house education and training to be targeted to specific projections that require optimisation, such as the HBL hip and knee.

Analysing the reasons for image rejection is an integral component of reject analysis. The most frequent reasons for image rejection in this study were ‘positioning’ error and ‘anatomy cut‐off’. This correlates with prior published literature analysing DR rejected images.^1^, ^2^, ^4^, ^5^ Previously with film‐screen radiography, exposure error was the most common reason for image rejection; however, this has shifted to positioning error and anatomy cut‐off with the introduction of CR and DR systems,^3^, ^4^, ^5^, ^10^, ^13^ as demonstrated in the current study. The prevalence of positioning errors as well as the considerable variation in radiographer reject rates indicates that the standards of image quality within the department may not be consistent. This is a common finding in the literature.^6^, ^7^, ^16^ A study by Dunn and Rogers^6^ found that radiologists are typically more lenient with image quality, accepting 50% of images that radiographers rejected due to positioning errors. Likewise, a study by Nol et al.^10^ suggested that one reason positioning error has become more common is due to the reduction in communication regarding image quality between radiologists and radiographers. Another potential reason for variations in reject rates between radiographers may be due to the differing years of clinical experience amongst participants. This indicates a need to establish more uniform standards of image quality as well as a feedback system within the DR medical imaging department to help guide in‐house education programs. Reject analysis is one example of an efficient and accurate way to gain such feedback and maintain these standards.

This study encountered some limitations worth consideration. Firstly, the method in which the data were collected and exported from the automatic reject analysis software could be improved. The data were not exported from the workstations on a regular basis and as a result, large portions of data were missing as a result of system upgrades or equipment breakdowns that necessitated the hard‐drive to be rebuilt. Evidently, this has implications for data reliability and it is recommended that future studies export the data from the workstations on a monthly basis and prior to scheduled system services. This will both reduce potential data loss as well as increase the regularity of feedback regarding department performance. Secondly, it was found that some rejected images were incorrectly categorised by radiographers; however, the reliability of image categorisation was unable to be assessed, as the software does not store the rejected images. In order to verify the reasons for image rejection, it would be beneficial to have access to the rejected images for a retrospective assessment. This could be achieved by sending the rejected images to a specific folder on the Picture Archiving and Communication System (PACS) or to an archive on an external drive. A further limitation of this study was that it was specific to the emergency imaging department. A future study worth conducting would be to repeat this audit with the inclusion of general radiographic reject rates. This would involve standardising the output of automatic reject analysis software on all workstations allowing a comparison to be made between the two different types of patient presentations within an emergency and general imaging department. This would produce a more comprehensive review of the entire medical imaging department performance.

A future study worthy of undertaking could involve an exposure index analysis. This would be advantageous because with DR, it is possible to overexpose a patient up to 5–10 times an average exposure without it being evident in the image, making it difficult to monitor patient exposure.^4^, ^15^, ^17^ For example, the current study found that underexposed images were rejected more frequently than overexposed images. In a study conducted by Zhang,^15^ underexposed images typically resulted in reduced image quality, whereas overexposed images are rewarded with high image quality, and radiographers therefore favour higher exposures, resulting in ‘dose creep’. In addition, Foos et al.^3^ explains that improved detector systems and post‐processing capabilities have contributed to the reduction in exposure errors with CR. This is an important factor as it has implications for patient dose and the radiographer's ability to ensure they are abiding by the ALARA principle.^4^, ^15^, ^18^ Current DR systems produce an exposure index or dose index for each image as a means of immediate feedback for the radiographer. The issue of over or underexposure could be easily addressed if developers of reject analysis software incorporate automatic recordings of exposure data for analysis.

## Conclusion

This investigation has addressed the research aims and found an average reject rate of 9% over the 15‐month study period. It was found that a single percentage was an inaccurate representation of department performance and deeper assessment of individual projections and radiographer reject rates was both necessary and an effective means to reduce reject rates and patient dose. The authors recommend that medical imaging departments develop uniform standards of image quality and utilise regular reject analysis as both a feedback and as an educational tool. Future studies into reject analyses specific to DR would benefit from collecting exposure index data in order to monitor and reduce patient exposure to ionising radiation.

## Conflict of Interest

The authors declare no conflict of interest.
